# Bayesian Self‐Optimization for Telescoped Continuous Flow Synthesis

**DOI:** 10.1002/anie.202214511

**Published:** 2022-12-13

**Authors:** Adam D. Clayton, Edward O. Pyzer‐Knapp, Mark Purdie, Martin F. Jones, Alexandre Barthelme, John Pavey, Nikil Kapur, Thomas W. Chamberlain, A. John Blacker, Richard A. Bourne

**Affiliations:** ^1^ Institute of Process Research and Development Schools of Chemistry & Chemical and Process Engineering University of Leeds Leeds LS2 9JT UK; ^2^ Institute of Process Research and Development School of Mechanical Engineering University of Leeds Leeds LS2 9JT UK; ^3^ IBM Research UK Daresbury Laboratory Daresbury WA4 4AD UK; ^4^ ISEL Pharmaceutical Technology and Development, Operations AstraZeneca Macclesfield UK; ^5^ Chemical Development Pharmaceutical Technology and Development, Operations AstraZeneca Macclesfield UK; ^6^ UCB Pharma SA All. de la Recherche 60 1070 Anderlecht Belgium

**Keywords:** Bayesian Optimization, Continuous Flow, Machine Learning, Medicinal Chemistry, Sustainable Chemistry

## Abstract

The optimization of multistep chemical syntheses is critical for the rapid development of new pharmaceuticals. However, concatenating individually optimized reactions can lead to inefficient multistep syntheses, owing to chemical interdependencies between the steps. Herein, we develop an automated continuous flow platform for the simultaneous optimization of telescoped reactions. Our approach is applied to a Heck cyclization‐deprotection reaction sequence, used in the synthesis of a precursor for 1‐methyltetrahydroisoquinoline C5 functionalization. A simple method for multipoint sampling with a single online HPLC instrument was designed, enabling accurate quantification of each reaction, and an in‐depth understanding of the reaction pathways. Notably, integration of Bayesian optimization techniques identified an 81 % overall yield in just 14 h, and revealed a favorable competing pathway for formation of the desired product.

## Graphical abstract

An autonomous continuous flow platform for the rapid development of multistep synthetic pathways is reported. New multipoint sampling and Bayesian optimization techniques were combined, enabling simultaneous identification of optimum reaction conditions within a pharmaceutical process. The short optimization times achieved are promising for development of telescoped reactions in the future.

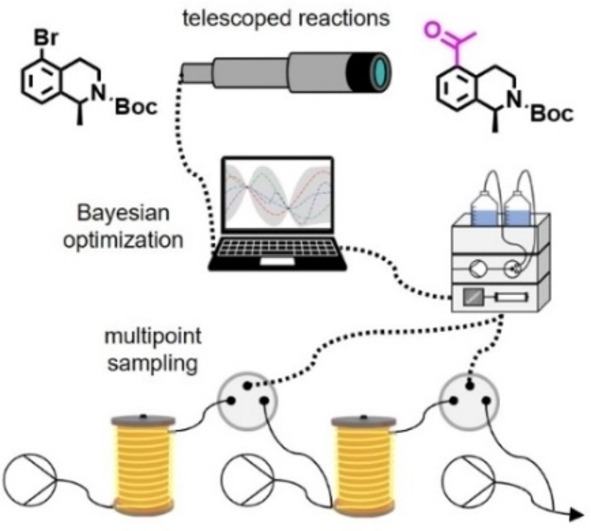

Active pharmaceutical ingredients (APIs) are traditionally synthesised in batchwise multistep sequences, involving iterative reaction‐workup‐purification‐isolation loops.^[1]^ Although functional, this approach suffers from long production times and potential supply chain disruptions. In contrast, continuous multistep synthesis benefits from in‐line purification and precise addition of reagents, resulting in more efficient uninterrupted reaction networks.[^2^, ^3^] This enables the flexible and on‐demand synthesis of APIs, in response to sudden changes in demand (e.g., pandemics).^[3a]^ However, as the structural complexity of small molecule APIs increases, so too does the need to simplify and optimize multistep syntheses where possible.

Reaction telescoping, where multiple transformations are achieved without the purification of intermediates, has the potential to significantly reduce solvent usage, which is estimated to account for 50 % of greenhouse gas emissions from the production of APIs.^[4]^ However, the task of optimizing telescoped reactions remains highly challenging. Concatenating steps not only increases the number of reaction variables, but also introduces complex interactions between the steps which must be considered holistically. For example, formation of an intermediate or by‐product from one reaction could have a negative influence on the downstream process (e.g., catalyst poisoning).^[5]^ This exemplifies that multistep syntheses cannot be realized by the simple combination of individually optimized reaction conditions, but rather requires all variables to be optimized simultaneously.^[6]^

Due to these increased complexities, development of telescoped reactions is currently a very resource and labour‐intensive task. Self‐optimizing systems, which combine flow reactors, inline/online analytics, and optimization algorithms, provide an autonomous method for accelerated and data‐enriched reaction development.^[7]^ However, application of these systems has been mostly limited to single step reactions with single objective,[^8^, ^9^, ^10^] multiobjective[^11^, ^12^, ^13^] or mixed variable[^14^, ^15^, ^16^] optimizations. Attempts to translate this approach to multistep syntheses were initially achieved utilising a single analytical measurement, at the end of the interconnected process.[^17^, ^18^, ^19^] Although successful in identifying a global optimum, sampling of only the process outlet severely limits the understanding of the individual steps, such as the formation and consumption of key intermediates. In addition, it becomes extremely difficult to relate the effect of each variable on the outcome of the reactions. Preferably, integration of multiple inline/online analytics enables monitoring of different chemical species across the multistep sequence, thus providing a more complete process understanding.^[20]^ For example, Jensen et al. utilized multiple inline FTIR and online LCMS instruments to monitor the optimization of a three‐step synthesis (S_*N*_Ar‐nitro reduction‐amide coupling) of sonidegib.^[5]^ Similarly, Kappe et al. utilized inline NMR and FTIR instruments to monitor the optimization of a two‐step synthesis (imine formation‐cyclization) of edaravone.^[21]^ However, quantification of reaction mixtures using inline spectroscopic methods requires additional chemometric modelling. This, combined with the substantial cost of using multiple analytical instruments, creates a significant barrier to the accessibility of this technology.

Herein, we report an automated continuous flow platform for the development of multistep syntheses, combining a Bayesian algorithm for simultaneous optimization of telescoped reactions, and a new multipoint sampling approach to maximise understanding of the reaction pathways (Figure 1A). To ensure this method is widely accessible, a multipoint sampling approach utilizing a single analytical instrument was needed. HPLC was selected as the analytical method, based on: (i) its facile ability to accurately quantify complex reaction mixtures, which are inherent in telescoped reactions due to a lack of intermediate purification; (ii) its widespread use in the pharmaceutical industry, where precise impurity profiling is required to meet the high regulatory standards. Inspired by daisy‐chaining, a method often used in electrical engineering to wire multiple devices in a sequence, we connected the HPLC in a loop with two 4‐port 2‐position sampling valves (Figure 1B). Multipoint sampling was then achieved by positioning the valves at the outlet of each reactor, and coding them to trigger sequentially (i.e., once the previous HPLC method had finished) within the optimization program. Although daisy‐chaining the valves together results in a variable HPLC dead volume, use of short lengths of capillary tubing prevented any noticeable dispersion or shifting of the analyte peaks between chromatograms.

**Figure 1:**
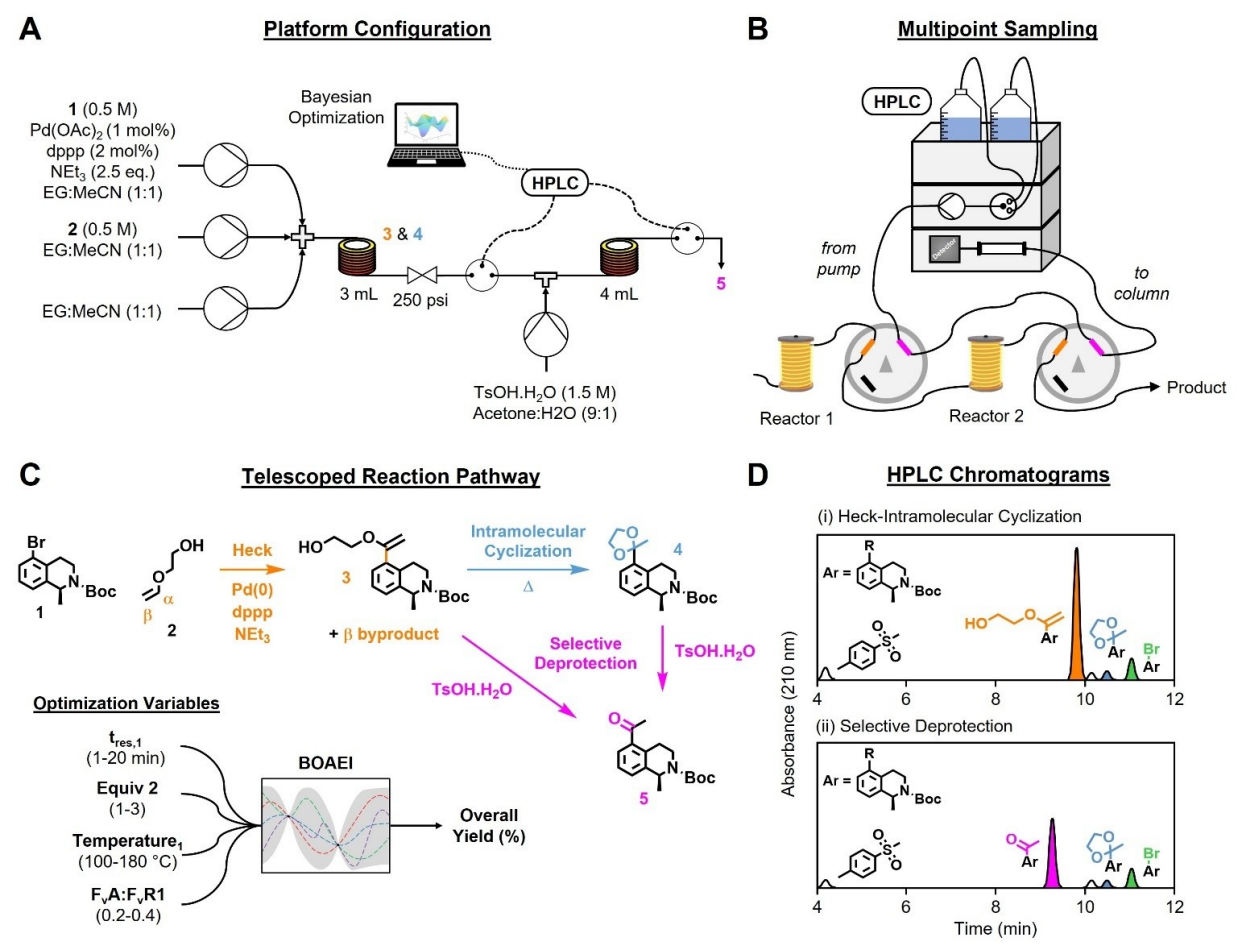
Telescoped process experiments for multistep synthesis of aryl ketone **5**. A) Platform configuration. B) Multipoint sampling approach using a single HPLC instrument. C) Telescoped reaction pathway and optimization variables, where: t_res,1_=residence time of first reactor; Equiv **2**=equivalents of ethylene glycol vinyl ether **2**; Temperature_1_=temperature of first reactor; F_V_A:F_V_R1=ratio of flow rate of acid (TsOH) to flow rate of first reactor. D) Example HPLC chromatograms from multipoint sampling i) first reactor: Heck coupling‐intramolecular cyclization; ii) second reactor: selective deprotection.

As the number of reactions in a process increases, so too does the number of optimizable variables, resulting in an exponential increase in the number of experiments required. Hence, to ensure this method remained practically viable, it was critical to integrate a state‐of‐the‐art optimization algorithm. Bayesian algorithms, which balance the exploration of areas of uncertainty with the exploitation of available information, have been applied as a tool for chemical reaction optimization in recent years.[^11^, ^22^, ^23^] Advantages of these methods include high robustness in the presence of experimental noise, and good optimization efficiency for objective functions that can be modelled well by either Gaussian processes,^[24]^ or other Bayesian models such as Bayesian neural networks,^[25]^ thus making them well suited for inherently expensive‐to‐evaluate experimental optimizations. Successful applications are typically limited to problems with fewer than 10–20 parameters,^[26]^ however this can be bypassed under certain circumstances where it is possible to execute reversibly compressive re‐featurization.^[27]^ Although this is not currently required due to the practical limits of experimental platforms, approaches such as this would need to be taken into consideration for longer and more complex multistep sequences as they develop in the future. In addition, the ability of these algorithms is significantly limited by the need to predefine the degree of trade‐off between exploration and exploitation; too much exploration is inefficient, and too much exploitation leads to initial biases and excessive local searching. To overcome this, we applied a Bayesian optimization algorithm with an adaptive expected improvement acquisition function (BOAEI), capable of dynamically controlling the explore/exploit trade‐off (see ESI for details).^[28]^

1‐Methyltetrahydroisoquinoline (1‐MeTHIQ) derivatives are of pharmaceutical interest for the treatment of depression.^[29]^ Thus, the telescoped synthesis of aryl ketone **5**, a potentially versatile precursor for 1‐MeTHIQ C‐5 functionalization, was chosen as an exemplary case study for autonomous multistep optimization.^[30]^ We envisaged the following reaction sequence: i) regioselective Pd‐catalyzed cross‐coupling between aryl bromide **1** and ethylene glycol vinyl ether **2**; ii) intramolecular cyclization of vinyl ether **3** to form ketal **4**; iii) selective acid‐catalyzed hydrolysis of ketal **4** to form aryl ketone **5** (Figure 1C). Initially, the viability of the proposed synthesis was assessed in batch, which also enabled isolation of each reaction component for HPLC method development and calibration (Figure 1D, see ESI for details).

Reported conditions using Pd(OAc)_2_/dppp in ethylene glycol (EG) at 145 °C, selectively converted aryl bromide **1** to ketal **4** in an 87 % yield over 2 h.^[31]^ Due to the poor solubility of bidentate phosphine ligands in pure EG, an EG:MeCN (1 : 1) solvent mixture was required to transition from batch to flow. Satisfyingly, full conversion of aryl bromide **1** was achieved at 175 °C in just 10 min, whilst retaining high selectivity for the α‐products (**3**=54 %; **4**=32 %). Inspired by the use of Amberlyst‐15 for the selective deprotection of *N*‐*tert*‐butyloxycarbonyl (*N*‐Boc) amino acetals, a range of solid acid catalysts were screened for the conversion of ketal **4** to aryl ketone **5**.^[32]^ Of those tested, only polymer‐bound tosylic acid (TsOH) exhibited any reactivity. This led to the use of TsOH⋅H_2_O in an acetone:H_2_O solvent mixture (9 : 1), which provided full conversion of ketal **4** over 69 h at room temperature, with complete selectivity for aryl ketone **5**. Attempts to reduce the reaction time, by increasing the temperature to 60 °C, resulted in Boc removal and a 50 % reduction in selectivity. Finally, to enable successful telescoping of the reactions, a constant excess of TsOH was required to quench NEt_3_ from the first step. Thus, only effective equivalents relative to the concentration of aryl bromide **1** are reported herein.

With the proposed multistep synthesis validated in batch, the automated flow platform was configured according to Figure 1A, and the optimization design space defined (Figure 1C, Optimization Variables). The BOAEI algorithm was initialized with nine Latin hypercube (LHC) experiments, and then allowed to run for 23 sequential iterations. Impressively, an optimum overall yield of 81 % was identified in just 13 total experiments, corresponding to a run time of 14 h (Figure 2A(i)). The algorithm demonstrated a good level of explore/exploit trade‐off, highlighted by most of the experiments exceeding the best result from the LHC, whilst still investigating less lucrative regions throughout. Optimal conditions corresponded to long residence times, high equivalents of **2**, moderate temperatures and low equivalents of TsOH (Figure 2A(ii)). Notably, the model identified the amount of TsOH to have significantly less influence on the overall yield compared to the other reaction conditions (Figure 2A(iii)).

**Figure 2:**
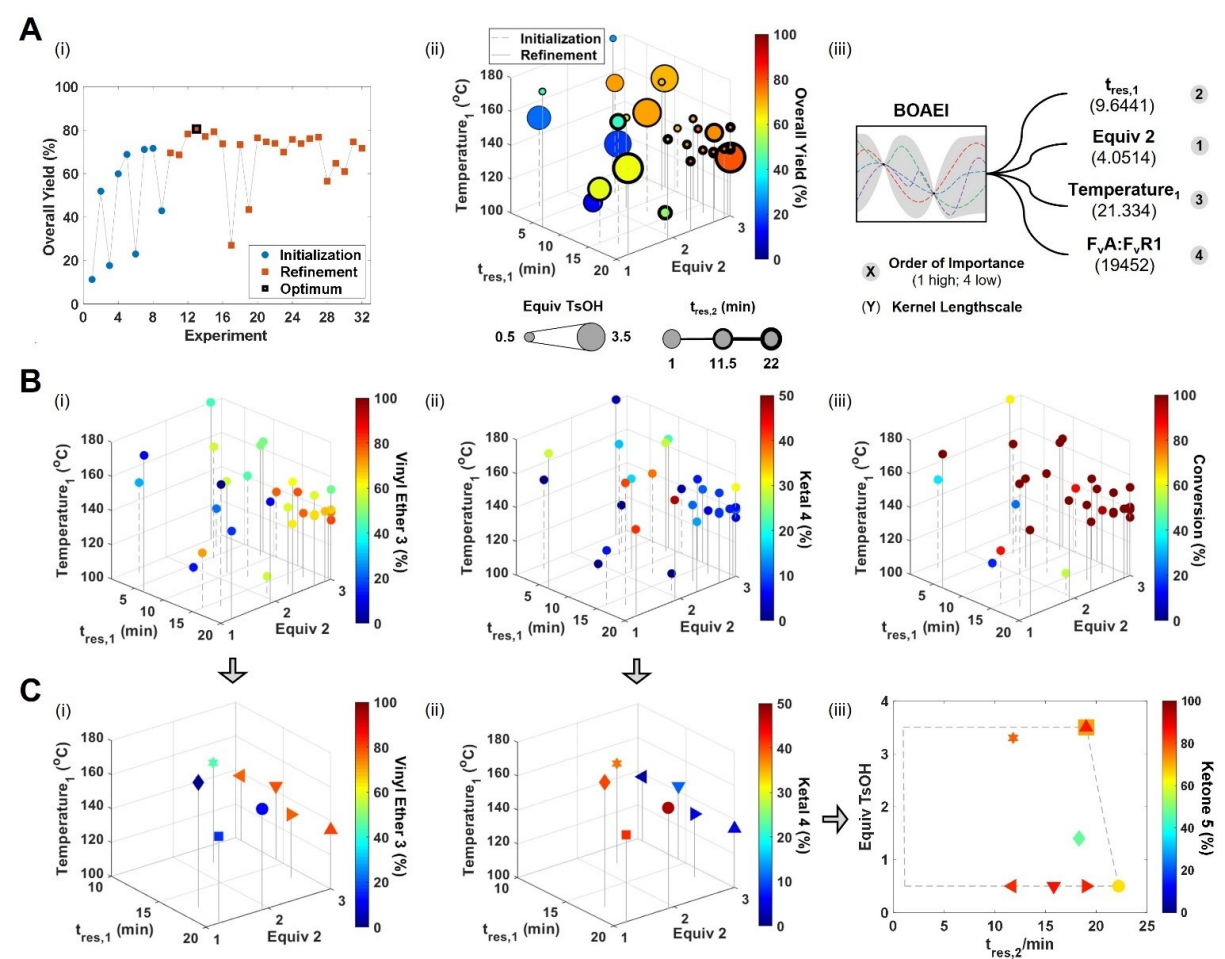
Self‐optimization results of the multistep synthesis. A) General optimization overview: i) optimization progress: overall yield versus experiment number; ii) scatter plot showing variables explored across the telescoped process; iii) model output showing relative importance of each variable. B) Reaction profiles for the Heck coupling‐intramolecular cyclization: i) yield of vinyl ether **3**; ii) yield of ketal **4**; iii) conversion of aryl bromide **1**. C) Reaction profiles for the selective deprotection: i) and ii) scatter plots showing eight datapoints, corresponding to the four best yielding reactions for vinyl ether **3** and ketal **4** respectively; iii) yield of aryl ketone **5** in the second step, where the shape of the datapoint corresponds to the same telescoped reaction (the constrained design space is highlighted by dashed lines).

Integration of multipoint sampling enabled detailed reaction profiles to be generated for each individual reaction step. The yield of vinyl ether **3** and ketal **4**, and the conversion obtained from the Heck coupling‐intramolecular cyclization step, are shown in Figure 2B(i,ii,iii) respectively. High conversions of aryl bromide **1** can be achieved under a wide range of conditions, resulting in different ratios of products **3** and **4**. Similar to the overall yield, formation of vinyl ether **3** is favored at long residence times (>14 min), high equivalents of **2** (> 2.5) and moderate temperatures (125‐140 °C), whereas significant amounts of ketal **4** only form at temperatures exceeding 140 °C. This suggests that the intramolecular cyclization has a relatively high activation energy, and that hydrolysis of vinyl ether **3** is instead the favorable pathway for formation of aryl ketone **5**. The latter can be confirmed by comparing the best yielding reactions for formation of vinyl ether **3** (Figure 2C(i)) and ketal **4** (Figure 2C(ii)) with the yield of the selective deprotection step (Figure 2C(iii)). Importantly, deprotection of mixtures where vinyl ether **3** was the major component gave greater than 83 % yield of aryl ketone **5**, independent of the hydrolysis conditions; correlating well with the models feature importance ranking. In contrast, deprotection of mixtures where ketal **4** was the major component required higher equivalents of TsOH to achieve aryl ketone **5** yields between 71 and 78 %.

In this case, telescoped flow optimization with multipoint sampling was critical for identifying the global optimum with complete process understanding. Monitoring of the first reaction was essential to observe the key vinyl ether **3** intermediate, due to its complete conversion under all subsequent hydrolysis conditions. Failure to observe this could have led to the incorrect conclusion that ketal **4** formation was favored under the optimum conditions. Accurate quantification of both reaction mixtures enabled the elucidation of different hydrolysis profiles for vinyl ether **3** and ketal **4**, and thus the identification of the favorable reaction pathway for the multistep synthesis of aryl ketone **5**. Indeed, if the Heck coupling‐intramolecular cyclization and selective deprotection steps had been optimized sequentially, targeting the formation of ketal **4** and aryl ketone **5**, an overall suboptimal process would have been developed (i.e., lower overall yield, higher temperatures and greater equivalents of acid required). In addition, the precise residence time and temperature control provided by continuous flow, were key in enabling the formation of vinyl ether **3** under productive conditions without significant cyclization.

In conclusion, we report an autonomous method for the optimization of multistep syntheses. Telescoped reactions are simultaneously optimized, to account for chemical interdependencies between the steps, and thus reduce the number of optimization campaigns required. Integration of a state‐of‐the‐art Bayesian algorithm allows identification of the global process optimum within a practical time frame. In addition, the multipoint HPLC sampling technique developed in this work is simple and widely accessible, enables monitoring and accurate quantification of each step, and thus provides an in‐depth understanding of the reaction pathways. Hence, this approach marks a conveniently accessible technology for accelerating the development of new multistep chemical syntheses.

## Conflict of interest

The authors declare no conflict of interest.

## Supporting information

As a service to our authors and readers, this journal provides supporting information supplied by the authors. Such materials are peer reviewed and may be re‐organized for online delivery, but are not copy‐edited or typeset. Technical support issues arising from supporting information (other than missing files) should be addressed to the authors.

Supporting Information

## Data Availability

The data that support the findings of this study are available in the Supporting Information of this article.
