# Supplementary material for: Bayesian Self‐Optimization for Telescoped Continuous Flow Synthesis
Source: Angew Chem Int Ed Engl. 2022 Dec 13;62(3):e202214511. doi: 10.1002/anie.202214511 (PMC10108149; doi:10.1002/anie.202214511)
Supplement: Supplementary file 1 — Supporting Information [file ANIE-62-0-s001.pdf]

Angewandte Chemie
Eine Zeitschrift der Gesellschaft Deutscher Chemiker

Supporting Information

Bayesian Self-Optimization for Telescoped Continuous Flow Synthesis

A. D. Clayton, E. O. Pyzer-Knapp, M. Purdie, M. F. Jones, A. Barthelme, J. Pavey, N. Kapur, T. W. Chamberlain, A. J. Blacker, R. A. Bourne**

Contents

1 Multistep Self-Optimization Platform 3
1.1 Automated Continuous Flow Reactor 3
1.2 Optimization Procedure 5
2 Experimental 8
2.1 Chemicals & Analytical Methods 8
2.2 Reaction Development 9
3 Telescoped Optimization 12
3.1 Reactor Setup 12
3.2 Results 13
4 Appendix 15
4.1 NMR Spectra 15
5 References 21

1 Multistep Self-Optimization Platform

1.1 Automated Continuous Flow Reactor

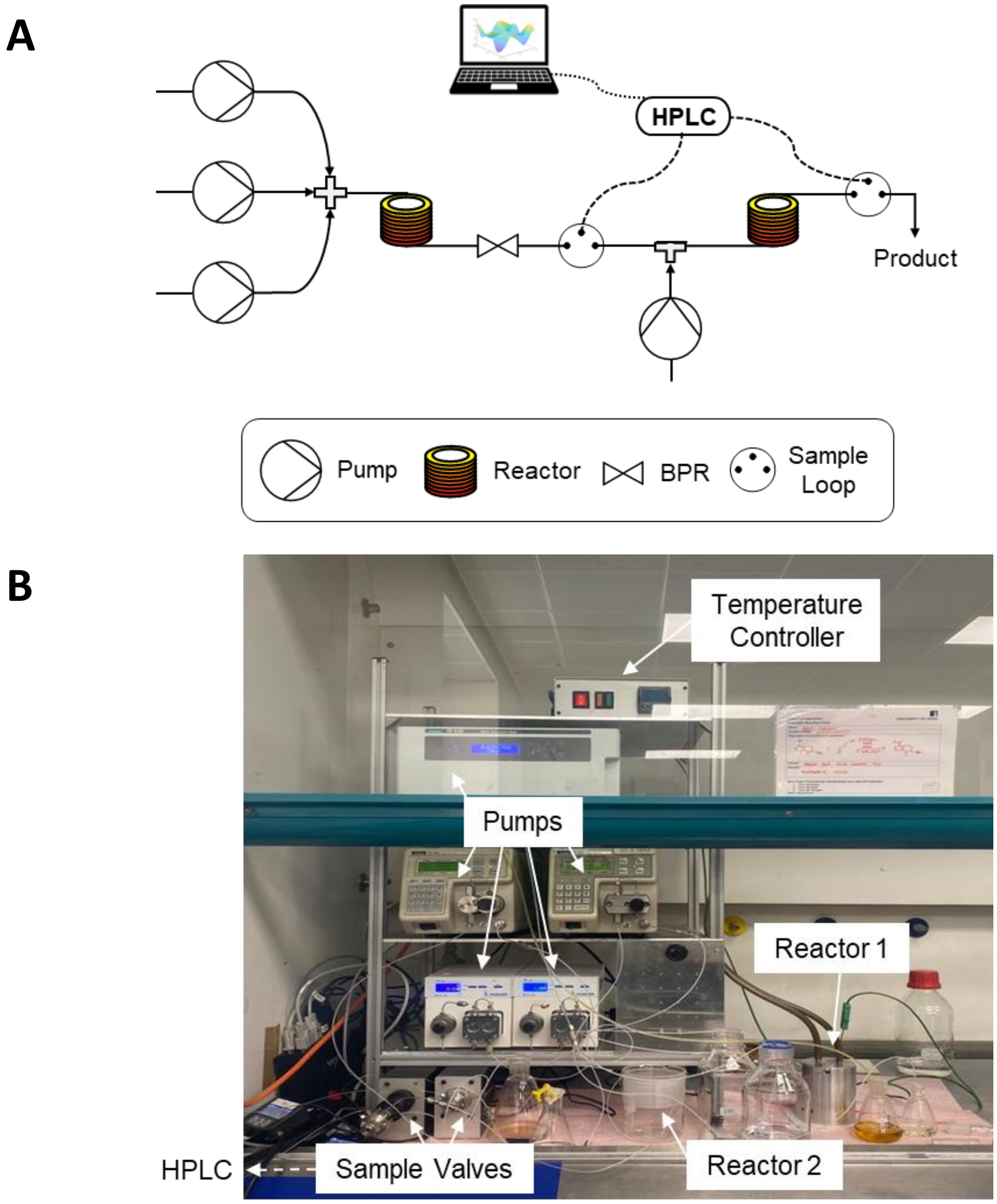

Scheme S1. Automated continuous flow reactor. (A) General schematic. BPR = back pressure regulator. (B) Labelled photo.

A

B

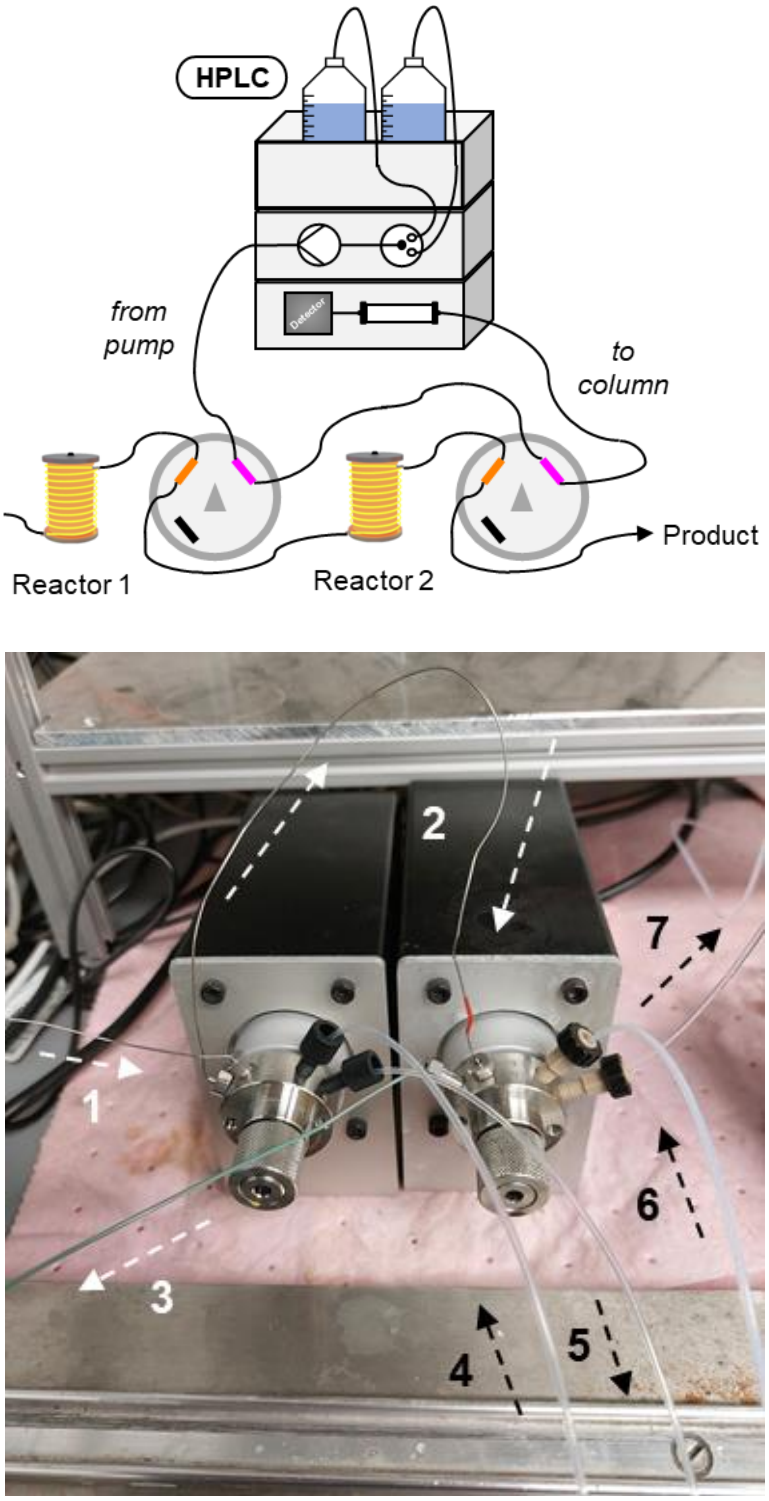

Figure S1. Multipoint sampling approach using a single HPLC instrument. (A) Diagram showing the daisy-chaining method. (B) Labelled photo of sampling valves showing all connections. White arrows = HPLC flow direction. **1** = from HPLC pump; **2** = valve one to valve two; **3** = to HPLC column. Black arrows = reaction mixture flow direction. **4** = from reactor one; **5** = to reactor two; **6** = from reactor two; **7** = to product collection.

Reagents were pumped using a combination of JASCO dual piston HPLC pumps (PU4185 semi-micro, PU-1580, PU-980) and pump streams were mixed using Swagelok SS-100-3 tee pieces. Tubular reactors of desired volume were made of either stainless steel or PFA tubing (1/16" OD, 1/32" ID), and fitted to custom-built cylindrical aluminium blocks. The temperature of the reactors was regulated using a feedback loop consisting of two nickel heating elements, a type K thermocouple and a Eurotherm 3200 temperature controller. The reactors were maintained under the desired fixed pressure using Upchurch Scientific back pressure regulators. Quantitative analysis was performed on an Agilent 1260 Infinity II HPLC instrument fitted with an Agilent Poroshell 120 EC-C18 reverse phase column (5 cm length, 4.6 mm ID, 2.7 μm particle size).

Online multipoint sampling was achieved by connecting the HPLC in a loop with two 4-port 2-position VICI EUDA-CI4W sample valves (0.5 μL injection volume) positioned at the outlet of each reactor (Figure S1). Each sample valve had two sets of inlet and outlet ports, one for the HPLC mobile phase and the other for the reaction mixture. The HPLC mobile phase flowed according to the following sequence: HPLC pump – valve one – valve two – HPLC column, where valves one and two were connected with an Agilent Capillary ST (0.17 × 250 mm, ≈ 23 μL). Similarly, the reaction mixture flowed according to the following sequence: reactor one – valve one – reactor two – valve two – product collection. Each valve could be triggered independently, enabling an aliquot of the reaction mixture exiting from reactor one or two to be introduced to the HPLC mobile phase for analysis. To avoid overlapping signals, the valves were scheduled to trigger sequentially with a time difference equal to that of the previous HPLC method.

1.2 Optimization Procedure

The Bayesian Optimizer with Adaptive Expected Improvement (BOAEI) algorithm was integrated with the automated flow reactor to enable closed-loop optimization of telescoped reactions (Scheme S1). The BOAEI algorithm is initialized with a space filling design, using $2n + 1$ (n = number of variables) Latin hypercube (LHC) sampling points, to provide sufficient exploratory information for use with the iterative process model. Within the algorithm, the surrogate model is constructed using Gaussian process regression (GPR), where a Gaussian process (GP) defines a distribution over all possible functions, *f(x)*, given the observed data. A GP is specified by a mean *m(x)* and covariance *k(x,x')* as follows:

$$f(x) \sim GP(m(x), k(x, x')) \tag{1}$$

$$m(x) = \mathbb{E}[f(x)] \tag{2}$$

$$k(x, x') = \mathbb{E}[(f(x) - m(x))(f(x') - m(x'))] \tag{3}$$

The prior over the distribution was defined with a mean of zero, and Z-score normalization of the input to the GP used to ensure this assumption holds true:

$$Z = \frac{x - \mu}{\sigma} \tag{4}$$

In defining the prior, a covariance function is also defined, which is used to calculate a similarity measure between two points. In this work, the Matérn 5/2 kernel was used:

$$k_{M:v=\frac{5}{2}}(r) = \left(1 + \frac{\sqrt{3}r}{l} + \frac{5r^2}{3l^2}\right) exp\left(-\frac{\sqrt{5}r}{l}\right) \tag{5}$$

After construction of the GP surrogate, an acquisition function is used to suggest the next sampling point. In this work, an adaptive expected improvement function was used, which dynamically controls the explore/exploit trade-off.[1] This is achieved by modifying the improvement which is implicitly tied to the underlying model, known as *contextual improvement*, or χ:

$$\chi = \frac{y_{pred} - f^* - c_v}{\sigma} \tag{6}$$

for maximization, where c_v is the contextual variance which can be written as:

$$c_v = \frac{\overline{\sigma^2}}{f^*} \tag{7}$$

where $\overline{\sigma^2}$ is the mean of the variances contained within the sampled posterior distribution.

An optimization program was written in MATLAB which controlled the pump flow rates, reactor temperature and sampling. For each iteration the reactor was allowed to stabilize at the desired operating temperature, the pumps were set to the required flow rates and left for two reactor volumes to reach steady state, and the sampling valves were triggered alongside HPLC analysis. To achieve multipoint sampling using a single HPLC instrument, the sampling valves were triggered sequentially, where the second valve was triggered after the first HPLC method had finished. To minimize the duration and material consumption per iteration: (i) pump flow rates were reduced to a minimum during heating/cooling of the reactor; (ii) initial LHC experiments were sorted in order of increasing temperature; (iii) sequential LHC experiments were started whilst analysis of the previous was running. The responses were calculated from the HPLC chromatograms, and used to update the surrogate model and generate the next set of reaction conditions using the BOAEI algorithm. The optimization was terminated after the maximum number of experiments was reached, which in this case was set based on the amount of starting materials available at the beginning of the optimization.

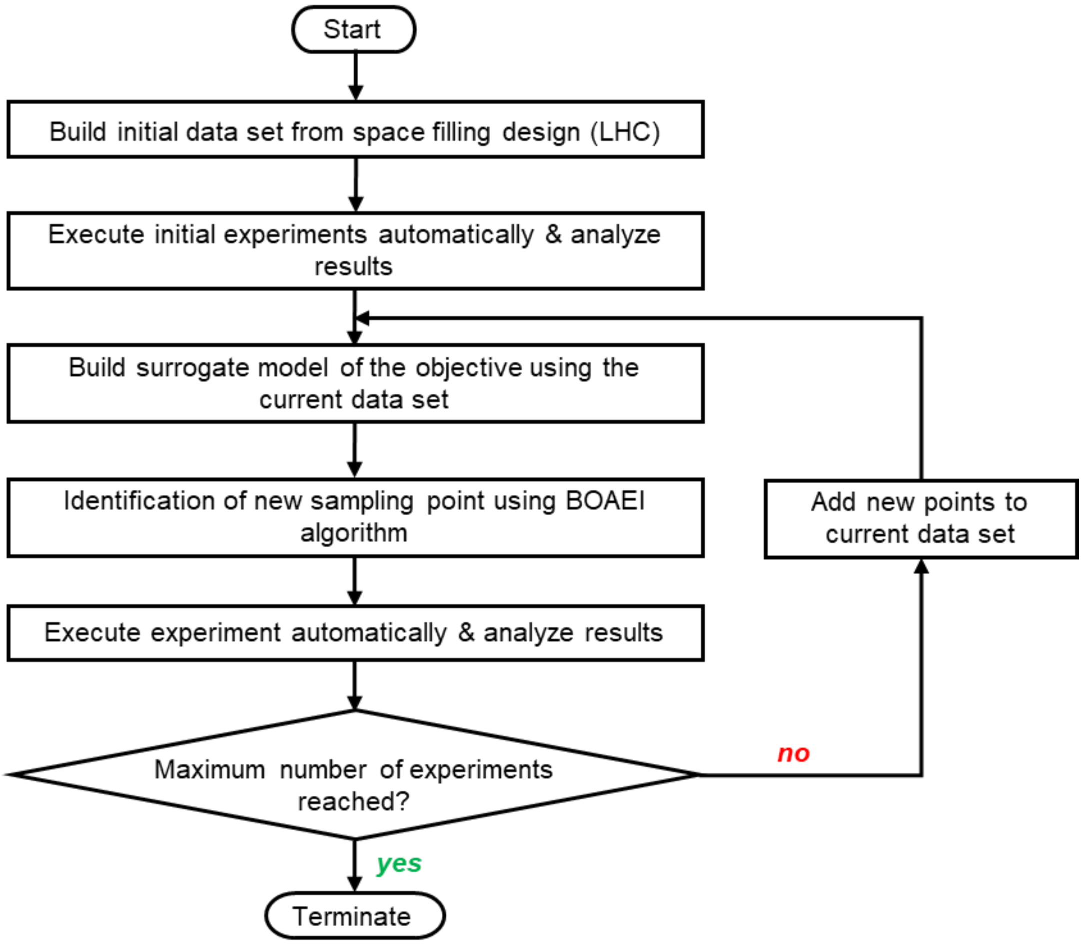

Figure S2. Flowchart of the optimization procedure.

2 Experimental

2.1 Chemicals & Analytical Methods

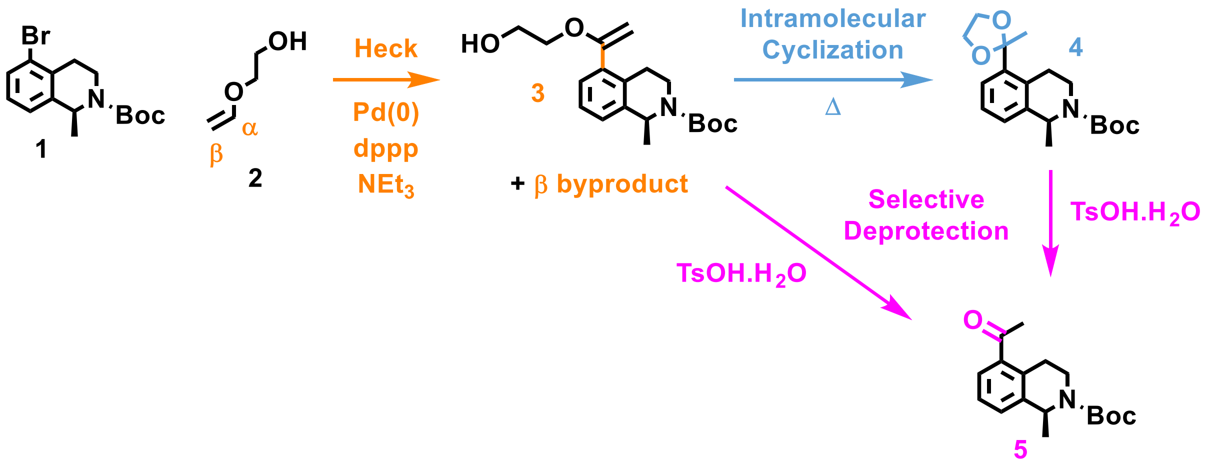

Scheme S2. Telescoped reaction pathway: (i) Heck reaction between aryl bromide **1** and ethylene glycol vinyl ether **2** to form vinyl ether **3**; (ii) intramolecular cyclization of vinyl ether **3** to form ketal **4**; (iii) selective deprotection of vinyl ether **3** and ketal **4** to form aryl ketone **5**.

Ethylene glycol vinyl ether **2** (97%, Sigma), palladium(II) acetate (>98%, Fluorochem), 1,3-bis(diphenylphosphino)propane (95%, Fluorochem), triethylamine (≥99.5%, Sigma), *p*-toluenesulfonic acid monohydrate (≥98%, Fluka), ethylene glycol (≥99%, Fisher), acetonitrile (≥99.9%, Sigma), acetone (≥99%, Sigma) and methyl *p*-tolyl sulfone (97%, Fluorochem) were purchased from suppliers and used without further purification. *tert*-butyl 5-bromo-(1*S*)-methyl-3,4-dihydro-1H-isoquinoline-2-carboxylate **1** was supplied by UCB Pharma, and was synthesized starting from *N*-(2-bromophenethyl)acetamide via a previously developed process.[2] Vinyl ether **3**, ketal **4** and aryl ketone **5** were synthesized and characterized during this work (see Section 2.2 and Appendix for details).

Characterization was performed using offline NMR spectroscopy and mass spectrometry. NMR spectroscopy was performed on a Bruker 400 UltraShield™ NMR Spectrometer (^{1}H NMR at 400 MHz, ^{13}C at 100 MHz) with the appropriate deuterated solvent. Chemical shifts in ^{1}H and ^{13}C NMR spectra and expressed as ppm downfield from TMS, and reported as singlet (s), doublet (d), triplet (t), quartet (q) and combinations thereof, or multiplet (m). Coupling constants (*J*) are quoted in Hz and are averaged between coupling partners. High resolution mass spectrometry was performed on a Bruker maXis Impact Mass Spectrometer using a Q-TOF detector with electrospray ionization in the positive mode.

Quantitative analysis was performed using online HPLC with multipoint sampling using methyl *p*-tolyl sulfone as an internal standard (see section 1 for details on multipoint sampling). HPLC analysis was performed on an Agilent 1260 Infinity II HPLC instrument fitted with an Agilent Poroshell 120 EC-C18 reverse phase column (5 cm length, 4.6 mm ID, 2.7 μm particle size). HPLC mobile phases were **A** H_2O (18.2 MΩ) and **B** MeOH. The method used was 16.3% **B** 2 min, 16.3 to 95.0% **B** 10 min, 95.0% **B** 1 min, 95.0 to 16.3% **B** 0.1 min, 16.3% **B** 0.9 min, flow rate 1.9 mL min^{-1}, column temperature 40 °C. In this case, the same method was used for both reaction steps, resulting in a total analysis time of 18 min for the telescoped process. Example chromatograms with retention times for key compounds are shown in Figure S3.

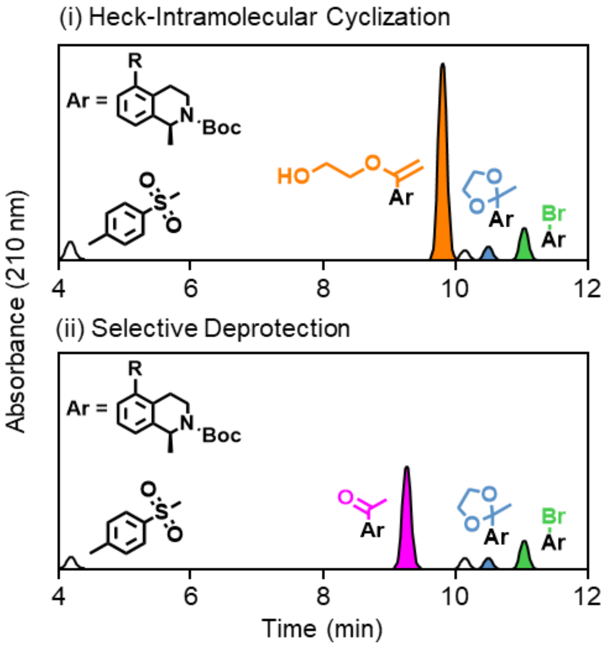

Figure S3. Typical HPLC chromatograms (i) Heck-intramolecular cyclization (step one); (ii) selective deprotection (step two). Retention times (min): methyl *p*-tolyl sulfone (internal standard) = 4.1; aryl ketone **5** = 9.3; vinyl ether **3** = 9.8; unidentified minor impurity = 10.1; ketal **4** = 10.4; aryl bromide **1** = 11.0.

2.2 Reaction Development

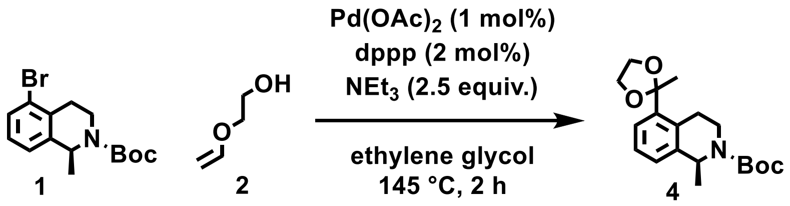

Scheme S3. Regioselective Pd-catalyzed cross-coupling between aryl bromide **1** and ethylene glycol vinyl ether **2**, with subsequent intramolecular cyclization to form ketal **4**.

An oven-dried round-bottom flask containing a stirrer bar was charged with aryl bromide **1** (1.63 g, 5 mmol), $Pd(OAc)_2$ (11.3 mg, 1 mol%), dppp (41.2 mg, 2 mol%), ethylene glycol (10 mL) and NEt_3 (1.74 mL, 12.5 mmol) sequentially under nitrogen at room temperature. The mixture was stirred and heated at 145 °C for 3-4 min, followed by addition of olefin **2** (1.35 mL, 15 mmol). The mixture was stirred at 145 °C for a further 2 hours before been cooled to room temperature.[3] Following addition of H_2O (30 mL), the aqueous phase was extracted with Et_2O (3 × 20 mL) and the combined organic layers washed with H_2O (20 mL), dried over Na_2SO_4 and concentrated *in vacuo*. The resultant mixture was passed through a silica gel filled Pasteur pipette using DCM as the eluent, which was then evaporated to provide ketal **4** in an 87% yield.

Ketal **4**: ^{1}H NMR ($CDCl_3$, 400 MHz) δ 7.38 (d, *J* = 7.5 Hz, 1H), 7.08 (t, *J* = 7.7 Hz, 1H), 6.99 (d, *J* = 6.9 Hz, 1H) 5.06 (d, *J* = 50.5 Hz, 1H), 4.00-3.59 (m, 5H), 3.24 (d, *J* = 36.6 Hz, 1H), 3.00 (m, 2H), 1.59 (s, 3H), 1.42 (s, 9H), 1.37 (d, *J* = 6.8 Hz, 3H); ^{13}C NMR ($CDCl_3$, 100 MHz) δ 154.5, 140.3, 140.0, 132.4, 127.2, 125.8, 124.4, 109.3, 79.5, 64.0, 51.4, 50.7, 39.2, 37.5, 28.6, 26.7, 22.7; *m/z* (ESI^+) $C_{19}H_{27}NO_4$ $[M+Na]^+$, calculated 356.1832, measured 356.1832.

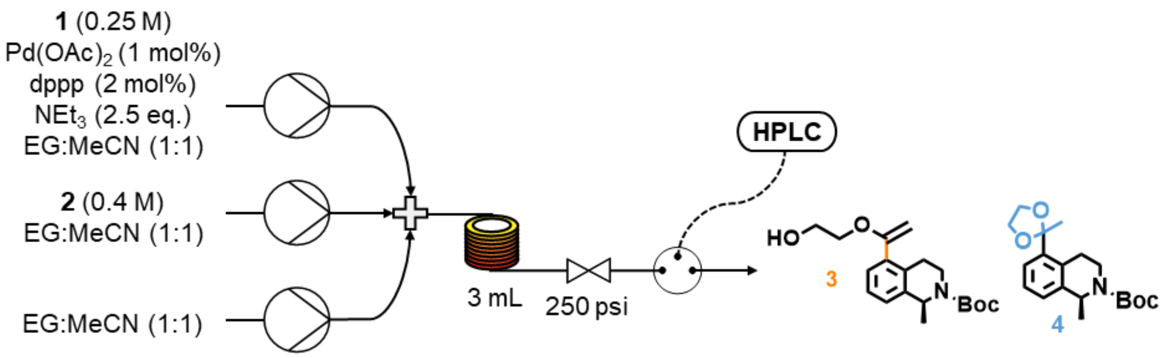

Scheme S4. Reactor setup for transitioning the Heck-intramolecular cyclization reaction from batch to flow. Reactor = stainless steel. For equipment specifications, see section 1.1.

Reservoir solutions were prepared by dissolving the desired reagents in solvent under stirring at ambient conditions. Pump 1: aryl bromide **1** (2.04 g, 6.25 mmol, 0.25 mol L^{-1}), $Pd(OAc)_2$ (14.03 mg, 1 mol%), dppp (51.56 mg, 2 mol%), NEt_3 (2.18 mL, 15.63 mmol, 0.625 mol L^{-1}) and methyl *p*-tolyl sulfone (0.43 g, 2.50 mmol, 0.10 mol L^{-1}) in EG/MeCN (1:1, 25 mL). Pump 2 = ethylene glycol vinyl ether **2** (0.90 mL, 10.0 mmol, 0.40 mol L^{-1}) in EG/MeCN (1:1, 25 mL). Pump 3 = EG/MeCN (1:1). The reactor was heated to 175 °C, and the flow rates set to achieve a 10 min residence time and 2.5 equivalents of **2** (F_v1 = 0.060 mL min^{-1}; F_v2 = 0.094 mL min^{-1}; F_v3 = 0.146 mL min^{-1}). The reactor was allowed to run for two reactor volumes to reach steady state, after which time the reaction mixture was analyzed via online HPLC. Full conversion was observed, and the yields of **3** and **4** were determined to be 54% and 32% respectively. An aliquot of the reaction mixture was collected from the outlet of the reactor, which was used to isolate vinyl ether **3** for calibration and characterization. Following addition of H_2O, the aqueous phase was extracted with Et_2O and the organic layer washed with H_2O, dried over Na_2SO_4 and concentrated *in vacuo*. Vinyl ether **3** was isolated from the crude product by flash chromatography on silica gel using a mixture of ethyl acetate and hexanes (3:2, 1% NEt_3) as the eluent.

Vinyl ether **3**: ^{1}H NMR ($CDCl_3$, 400 MHz) δ 7.16-7.01 (m, 3H), 5.10 (d, *J* = 48.5 Hz, 1H), 4.29 (d, *J* = 2.3 Hz, 1H), 4.14 (d, *J* = 2.3 Hz, 1H), 3.88-3.80 (m, 4H), 3.09 (d, *J* = 44.1 Hz, 1H), 2.84 (s, 1H), 2.70 (dt, *J* = 16.6, 3.7 Hz, 1H), 1.99 (s, 1H), 1.41 (s, 9H), 1.37 (d, *J* = 6.8 Hz, 3H); ^{13}C NMR ($CDCl_3$, 100 MHz) δ 161.3, 154.5, 139.1, 138.7, 137.5, 132.9, 127.3, 125.8, 86.4, 79.7, 69.0, 60.9, 50.8, 50.1, 38.2, 36.7, 28.5, 27.0, 22.2; *m/z* (ESI$^+$) $C_{19}H_{27}NO_4$ $[M+Na]^+$, calculated 356.1832, measured 356.1837.

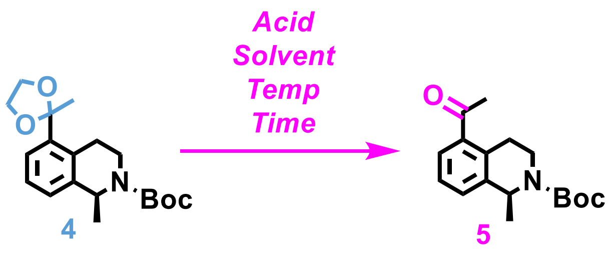

Scheme S5. Selective acid-catalyzed hydrolysis of ketal **4** to form aryl ketone **5**. Specific reaction conditions (acid, solvent, temp, time) are provided in Table S1.

General procedure for solid acid catalysts (entries 1-5): To a solution of ketal **4** (200 mg) in the solvent (25 mL) was added 50 μL of H_2O and 50 mg of the acid. The reaction mixture was stirred at the desired temperature for the specified reaction time. The crude product was filtered, concentrated *in vacuo,* and analyzed by ^{1}H NMR.

General procedure for polymer-bound TsOH (entries 6-7): To a solution of ketal **4** (150 mg) in the solvent (20 mL) was added polymer-bound TsOH (0.5 g, 1-2 mmol g^{-1}). The reaction mixture was stirred at the desired temperature for the specified reaction time. The crude product was filtered, concentrated *in vacuo*, and analyzed by ^{1}H NMR. Entry 6 was purified by flash column chromatography on silica gel using a mixture of ethyl acetate and hexanes (3:2, 1% NEt_3) as eluent.

General procedure for $TsOH{\cdot}H_2O$ (entries 8-12): To a solution of ketal **4** (240 mg, 0.72 mmol) in the solvent (20 mL) was added $TsOH{\cdot}H_2O$ (164.3 mg, 0.86 mmol). The reaction mixture was stirred at the desired temperature for the specified reaction time. The reaction mixture was then quenched with K_2CO_3 (118.9 mg, 0.86 mmol) and the majority of the solvent removed *in vacuo*. The residue was redissolved in H_2O (20 mL) and extracted with DCM (3 × 20 mL), washed with H_2O (20 mL), dried over Na_2SO_4 and concentrated *in vacuo*.

Table S1. Screening conditions for selective deprotection of a ketone in the presence of a Boc-protected amine (Scheme S5). Imps = by-product impurities.

Entry	Acid	Solvent	T/°C	Time/h	Conv/%[a]	Yield/%[a]	Imps?
1	Amberlyst 15	Acetone	25	18	n.r.	-	✗
2	Amberlyst 15	MeCN	82	18	n.r.	-	✗
3	Amberlite 120	MeCN	82	22	n.r.	-	✗
4	D521 Nafion	MeCN	25	20	n.r.	-	✗
5	Nafion NR50	MeCN	82	26	n.r.	-	✗
6	TsOH (polymer-bound)	Acetone:H_2O (9:1)	25	5.5	45	45 (39[b])	✗
7	TsOH (polymer-bound)	Acetone:H_2O (1:1)	60	17.5	100	0	✓
8	$TsOH{\cdot}H_2O$ (0.05 eq.)	Acetone	25	3	30	0	✓
9	$TsOH{\cdot}H_2O$ (0.10 eq.)	MeCN	25	5	n.r.	-	✗
10	$TsOH{\cdot}H_2O$ (1.20 eq.)	Acetone:H_2O (9:1)	25	5.5	61	61	✗
11	$TsOH{\cdot}H_2O$ (1.20 eq.)	Acetone:H_2O (9:1)	60	19	100	50	✓
12	$TsOH{\cdot}H_2O$ (1.20 eq.)	Acetone:H_2O (9:1)	25	69	100	100 (93[b])	✗

[a] Determined by ^{1}H NMR post work-up. [b] Isolated yield.

<u>Aryl Ketone **5**:</u> ^{1}H NMR ($CDCl_3$, 400 MHz) δ 7.49 (t, *J* = 4.4 Hz, 1H), 7.20 (d, *J* = 1.7 Hz, 1H), 7.19 (d, *J* = 1.5 Hz, 1H), 5.11 (d, *J* = 66.8 Hz, 1H), 3.96 (d, *J* = 48.6 Hz, 1H), 3.18-2.90 (m, 3H), 2.50 (s, 3H), 1.42 (s, 9H), 1.38 (d, *J* = 6.8 Hz, 3H); ^{13}C NMR ($CDCl_3$, 100 MHz) δ 201.9, 154.3, 134.5, 130.8, 128.5, 128.1, 127.5, 125.7, 79.8, 66.7, 40.6, 29.8, 28.5, 27.4, 21.8; *m/z* (ESI^+) $C_{17}H_{23}NO_3$ $[M+H]^+$, calculated 290.1751, measured 290.1748; $[M+Na]^+$, calculated 312.1570, measured 312.1567.

3 Telescoped Optimization

3.1 Reactor Setup

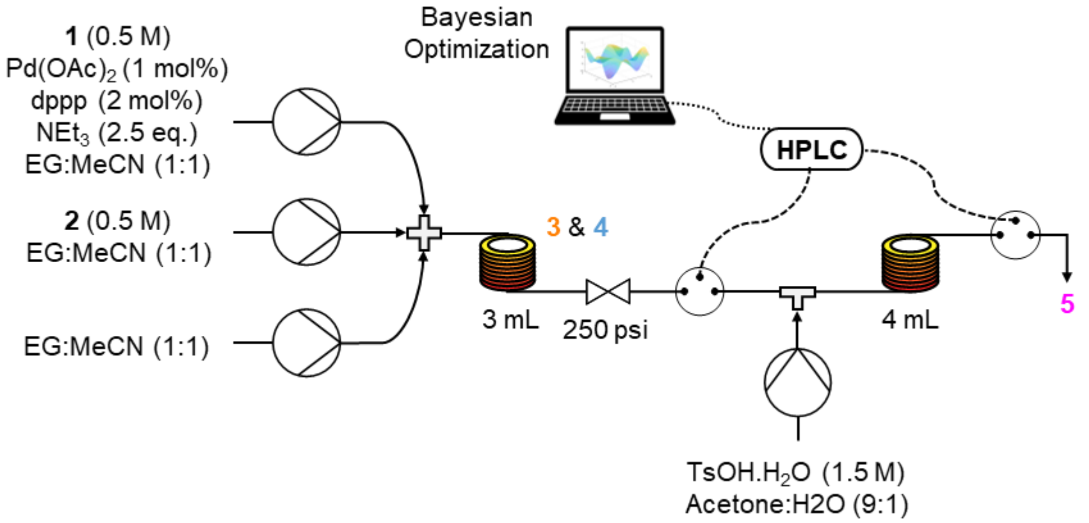

Scheme S6. Reactor setup for automated optimization of the telescoped reaction sequence (Heck-cyclization-deprotection). Reactor 1 = stainless steel; reactor 2 = PFA. For equipment specifications, see section 1.1.

The self-optimization was conducted with respect to four variables: residence time of the first reactor ($t_{res,1}$), equivalents of ethylene glycol vinyl ether **2**, temperature of the first reactor ($Temperature_1$) and ratio of flow rate of acid to flow rate of the first reactor ($F_VA:F_VR1$, related to the equivalents of TsOH). The lower and upper bounds for each variable are shown in Figure S4. Fixed parameters were the concentration of aryl bromide **1** in the first reactor (0.1 M), and the temperature of the second reactor (room temperature, Table S1 entry 11 *cf*. entry 12). The objective of the optimization was to maximize the overall yield of aryl ketone **5**, using the BOAEI algorithm (see section 1.2 for details).

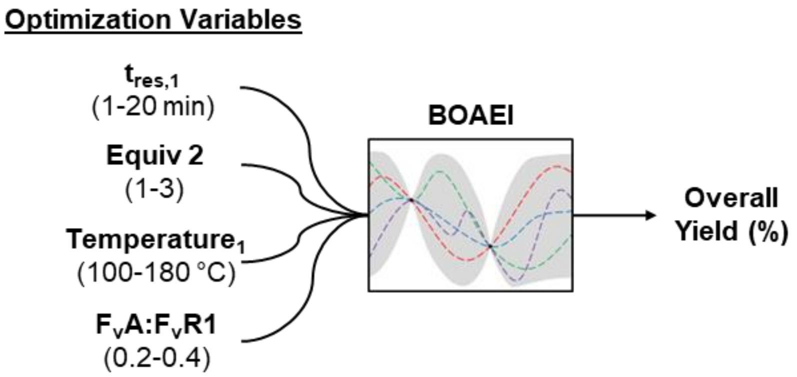

Figure S4. Reaction variables and the defined search space for the telescoped optimization.

Reservoir solutions were prepared by dissolving the desired reagents in solvent under stirring at ambient conditions. Pump 1: aryl bromide **1** (16.31 g, 50 mmol, 0.50 mol L^{-1}), $Pd(OAc)_2$ (112.3 mg, 1 mol%), dppp (412.4 mg, 2 mol%), NEt_3 (17.42 mL, 125 mmol, 1.25 mol L^{-1}) and methyl *p*-tolyl sulfone (1.70 g, 10 mmol, 0.10 mol L^{-1}) in EG/MeCN (1:1, 100 mL). Pump 2 = ethylene glycol vinyl ether **2** (4.49 mL, 50 mmol, 0.50 mol L^{-1}) in EG/MeCN (1:1, 100 mL). Pump 3 = EG/MeCN (1:1). Pump 4: $TsOH{\cdot}H_2O$ (28.53 g, 150 mmol, 1.5 mol L^{-1}) in acetone/H_2O (9:1, 100 mL).

3.2 Results

Table S2. Optimization results with respect to the first step (Heck-intramolecular cyclization). $t_{res,1}$ = residence time of first reactor; Equiv **2** = equivalents of ethylene glycol vinyl ether; $Temp_1$ = temperature of first reactor; Conv = conversion of aryl bromide **1**; Overall Yield = yield of aryl ketone **5** across all steps.

Entry	$t_{res,1}$/min	Equiv 2	$Temp_1$/°C	Conv/%	3/%	4/%	Overall Yield/%
1	7.6	1.8	105.5	15.9	13.3	0.2	11.3
2	18.5	2.0	112.6	56.6	57.7	0.2	51.9
3	3.9	2.6	124.4	23.3	22.0	0.0	17.7
4	14.9	1.2	131.7	86.2	71.3	7.1	59.9
5	10.5	3.0	138.1	86.4	77.0	2.0	68.8
6	2.0	1.4	152.5	35.3	29.5	0.0	23.0
7	12.2	2.3	158.8	99.9	44.1	38.0	71.1
8	5.4	2.4	165.2	98.9	56.9	15.8	71.6
9	17.2	1.3	173.5	99.9	0.4	40.4	42.9
10	8.2	3.0	164.3	99.9	48.2	22.1	69.5
11	8.7	2.9	164.1	99.7	48.8	28.5	68.6
12	14.2	3.0	143.1	99.5	78.5	10.9	78.2
13	20.0	3.0	139.8	99.8	76.2	13.0	80.5
14	20.0	2.3	141.4	99.9	63.5	14.2	77.0
15	20.0	3.0	134.6	99.8	81.1	5.0	79.2
16	20.0	3.0	152.5	100.0	48.1	30.6	73.7
17	1.0	2.8	180.0	63.4	43.3	2.5	27.0
18	17.1	3.0	145.1	99.8	59.3	8.9	73.3
19	6.7	1.0	180.0	100.0	11.4	28.8	43.4
20	19.5	3.0	139.1	100.0	67.2	11.0	76.5
21	20.0	3.0	139.3	99.6	75.6	8.3	74.6
22	20.0	3.0	140.7	99.9	66.6	6.6	73.9
23	13.3	3.0	147.8	99.9	62.2	7.2	69.9
24	20.0	2.7	141.0	99.9	66.0	7.8	75.7
25	17.1	2.8	137.3	95.1	77.9	4.9	73.8
26	20.0	2.7	141.8	99.9	64.5	8.7	76.1
27	20.0	2.7	141.6	99.9	65.1	8.6	76.7
28	20.0	1.9	159.5	100.0	11.8	47.5	56.5
29	9.5	2.2	153.2	99.9	56.4	16.9	64.7
30	20.0	1.2	151.4	100.0	16.8	41.5	60.9
31	19.9	2.9	140.9	99.8	68.3	7.5	74.6
32	17.3	2.5	144.8	100.0	59.0	11.2	71.6

Table S3. Optimization results with respect to the second step (selective deprotection). $F_vA:F_vR1$ = ratio of flow rate of acid (TsOH) to flow rate of first reactor; $t_{res,2}$ = residence time of second reactor; Overall Yield = yield of aryl bromide **5** across all steps.

Entry	$F_VA:F_vR1$	$t_{res,2}$/min	Equiv TsOH	5/%	Overall Yield/%
1	0.298	7.8	2.0	71.7	11.3
2	0.235	20.0	1.1	94.2	51.9
3	0.377	3.7	3.2	77.7	17.7
4	0.333	14.9	2.5	74.9	59.9
5	0.199	11.7	0.5	83.4	68.8
6	0.345	2.0	2.7	71.9	23.0
7	0.384	11.8	3.3	77.8	71.1
8	0.285	5.6	1.8	78.6	71.6
9	0.259	18.3	1.4	47.1	42.9
10	0.377	7.9	3.2	75.5	69.5
11	0.201	9.7	0.5	76.3	68.6
12	0.199	15.8	0.5	85.9	78.2
13	0.200	22.2	0.5	88.3	80.5
14	0.200	22.2	0.5	82.9	77.0
15	0.400	19.0	3.5	84.9	79.2
16	0.200	22.2	0.5	81.6	73.7
17	0.200	1.1	0.5	49.1	27.0
18	0.280	17.9	1.7	80.3	73.3
19	0.202	7.5	0.5	51.9	43.4
20	0.201	21.6	0.5	90.5	76.5
21	0.200	22.2	0.5	82.7	74.6
22	0.200	22.2	0.5	80.5	73.9
23	0.200	14.8	0.5	76.9	69.9
24	0.200	22.2	0.5	82.2	75.7
25	0.200	19.0	0.5	84.5	73.8
26	0.200	22.2	0.5	83.0	76.1
27	0.200	22.2	0.5	83.8	76.7
28	0.200	22.2	0.5	64.9	56.5
29	0.199	10.6	0.5	77.4	64.7
30	0.400	19.0	3.5	71.7	60.9
31	0.199	22.1	0.5	81.9	74.6
32	0.202	19.2	0.5	79.4	71.6

4 Appendix

4.1 NMR Spectra

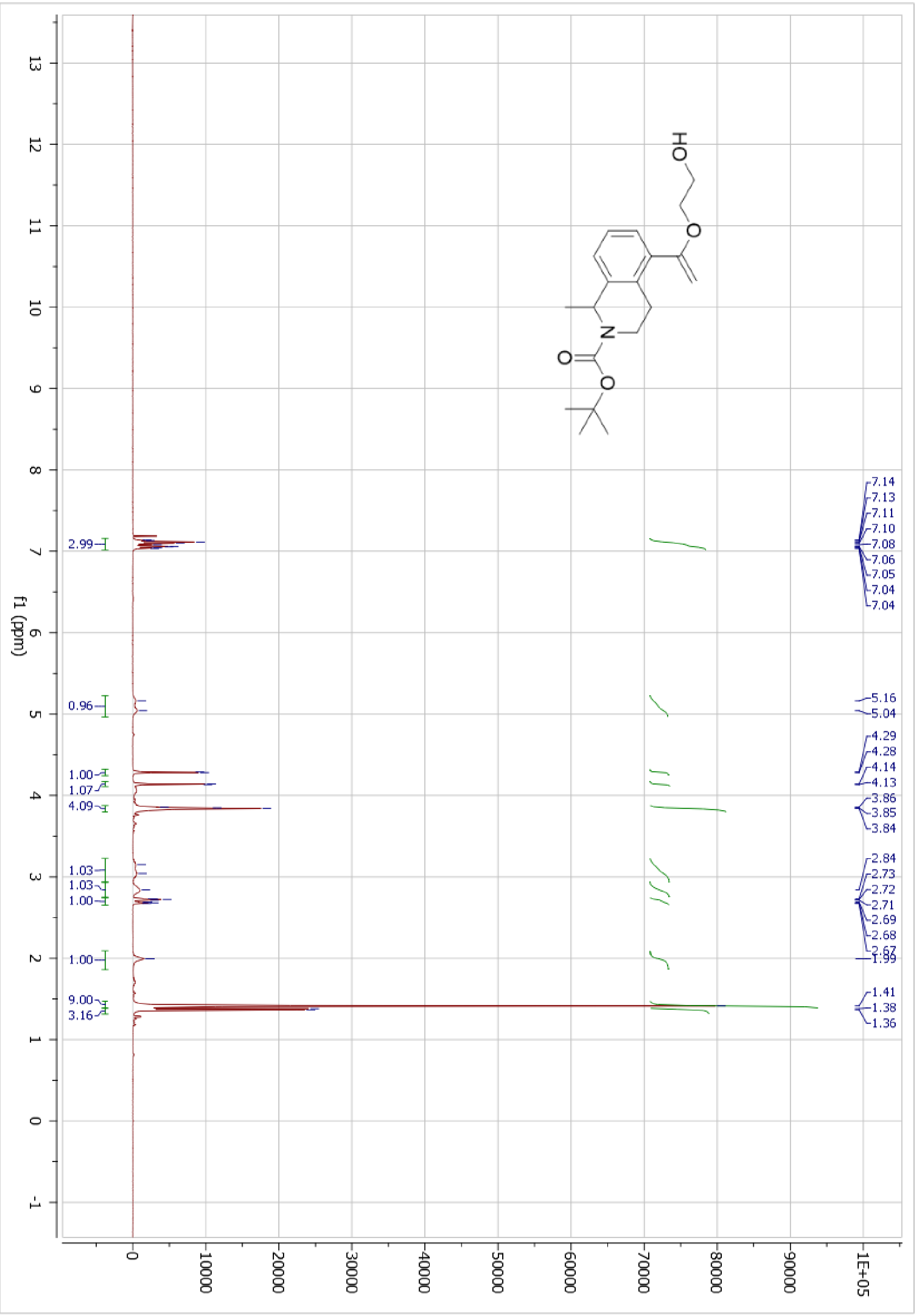

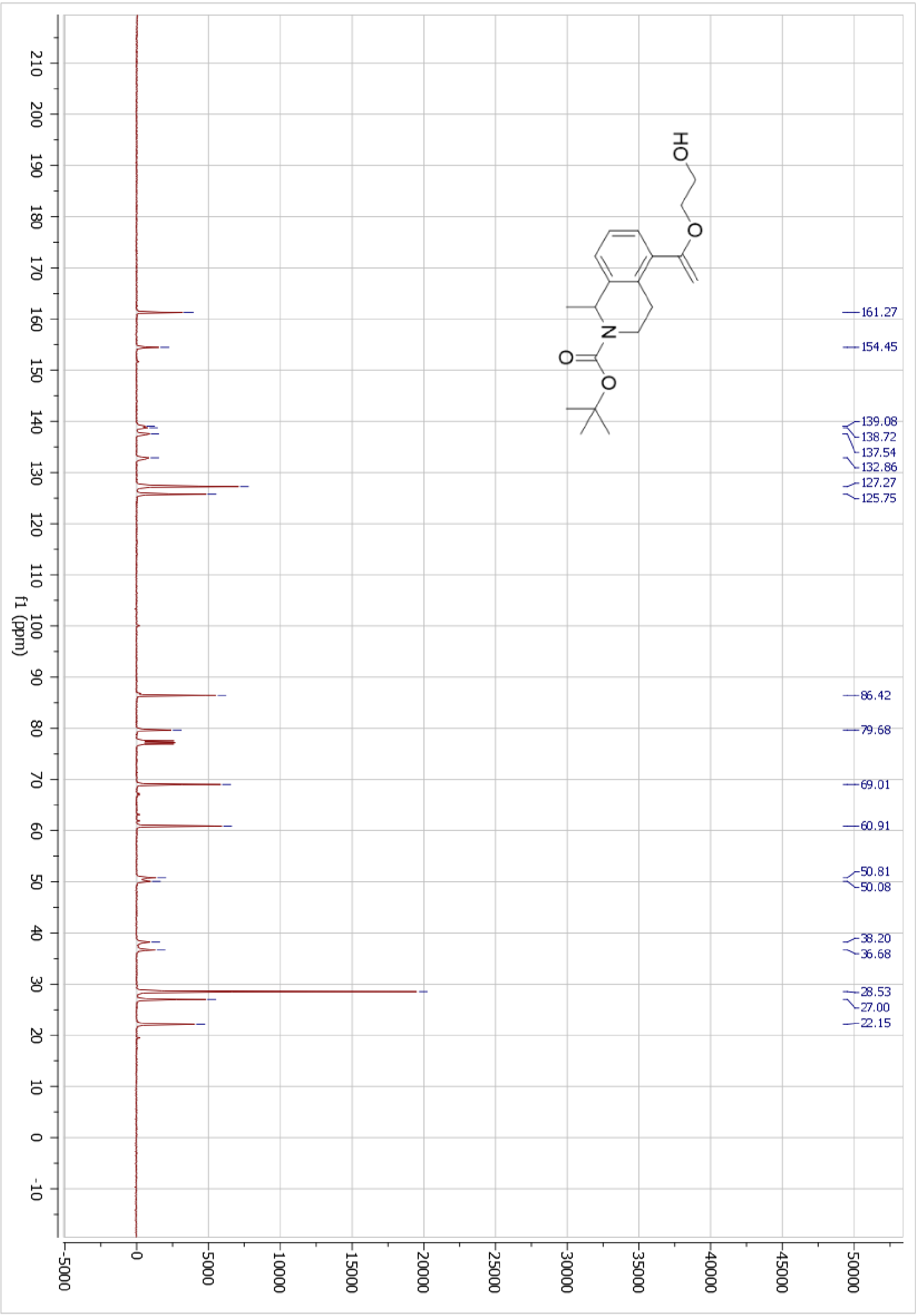
161.27
154.45
139.08
138.72
137.54
132.86
127.27
125.75
86.42
79.68
69.01
60.91
50.81
50.08
38.20
36.68
28.53
27.00
22.15
f1 (ppm)
HO
O
N
O
O

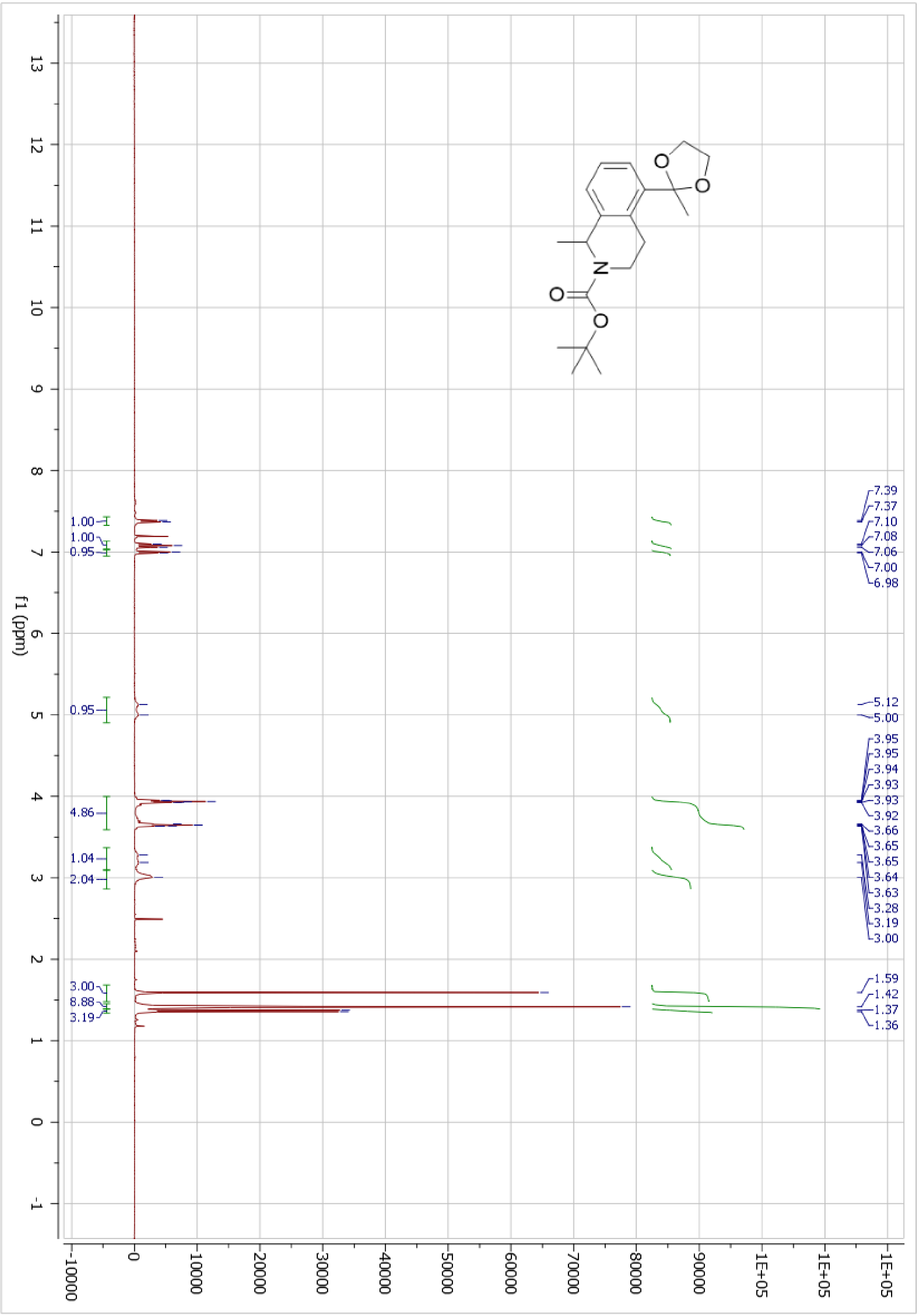
7.39
7.37
7.10
7.08
7.06
7.00
6.98
5.12
5.00
3.95
3.95
3.94
3.93
3.93
3.92
3.66
3.65
3.65
3.64
3.63
3.28
3.19
3.00
1.59
1.42
1.37
1.36
1.00
1.00
0.95
0.95
4.86
1.04
2.04
3.00
8.88
3.19
f1 (ppm)

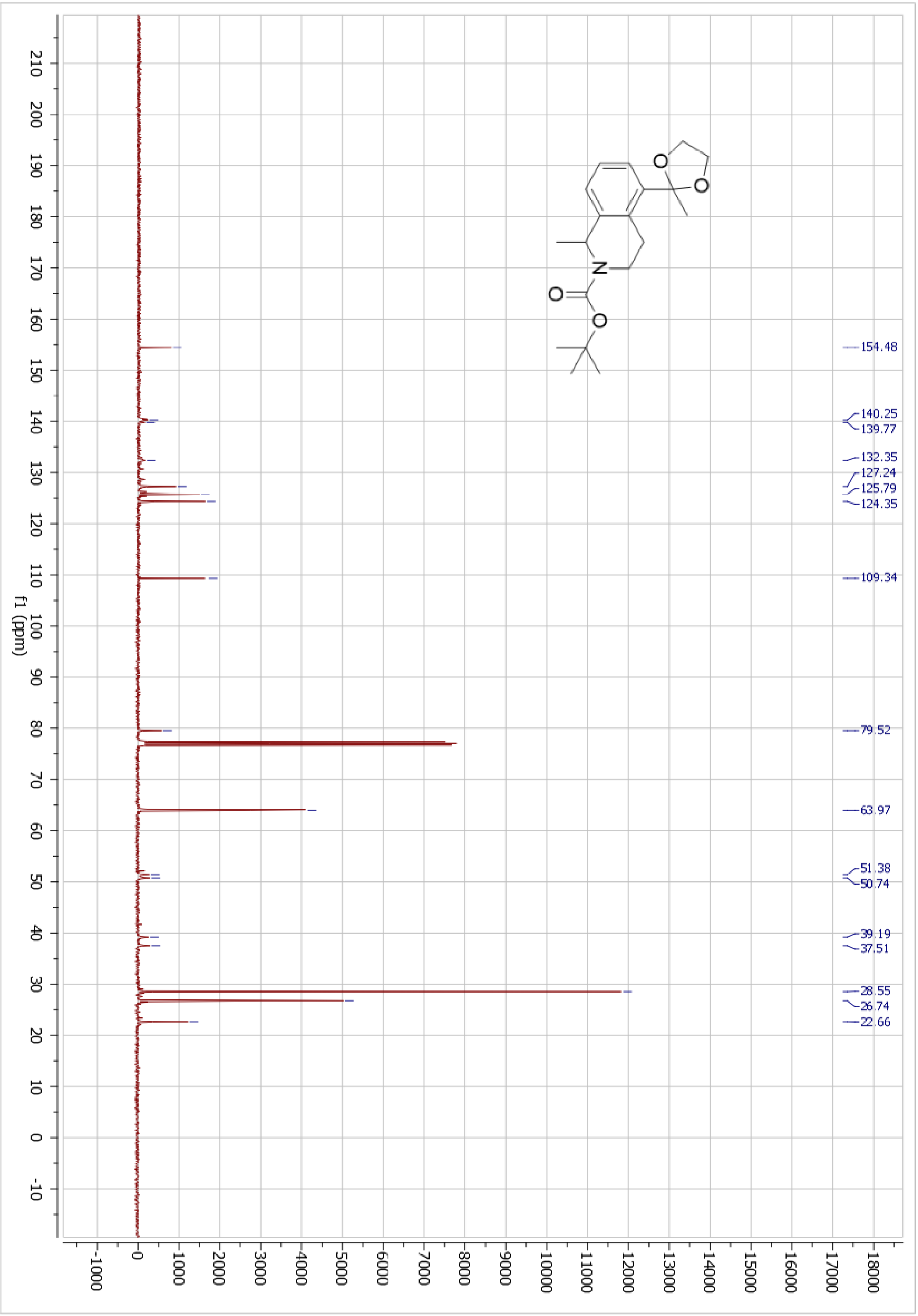
154.48
140.25
139.77
132.35
127.24
125.79
124.35
109.34
79.52
63.97
51.38
50.74
39.19
37.51
28.55
26.74
22.66
f1 (ppm)

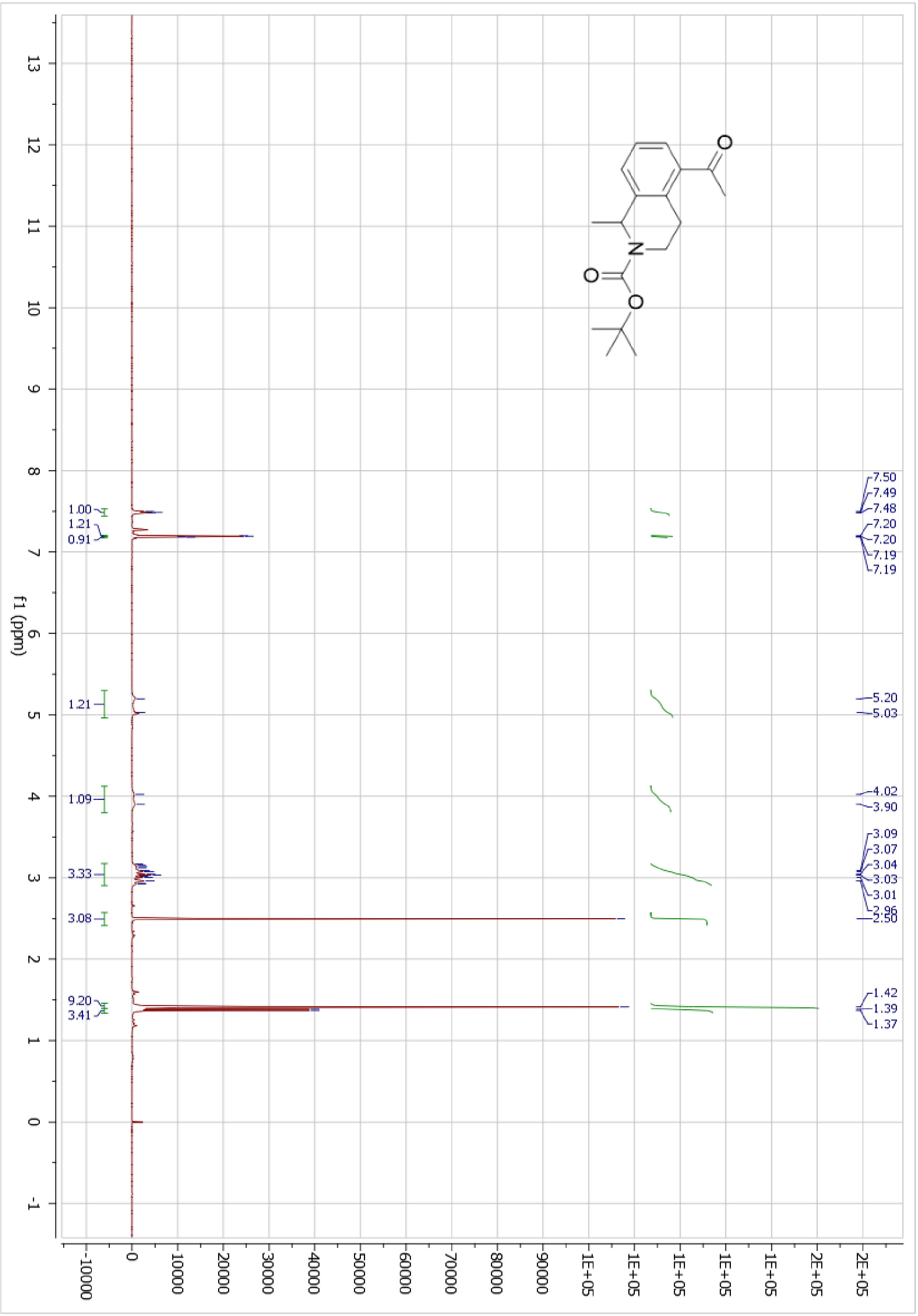

7.50
7.49
7.48
7.20
7.20
7.19
7.19
5.20
5.03
4.02
3.90
3.09
3.07
3.04
3.03
3.01
2.96
2.50
1.42
1.39
1.37
1.00
1.21
0.91
1.21
1.09
3.33
3.08
9.20
3.41
f1 (ppm)

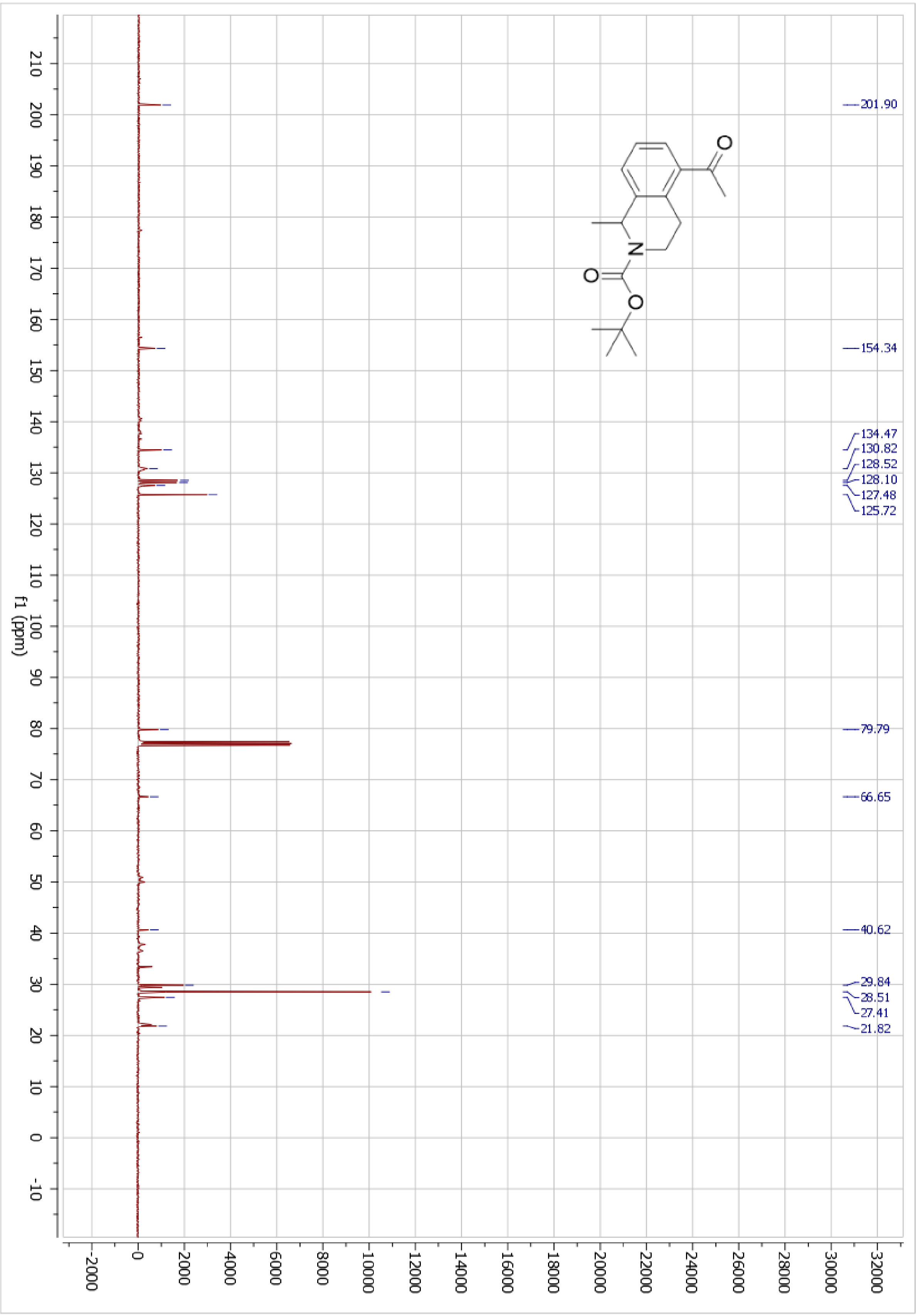

201.90
154.34
134.47
130.82
128.52
128.10
127.48
125.72
79.79
66.65
40.62
29.84
28.51
27.41
21.82
f1 (ppm)

5 References

[1] D. Jasrasaria, E. O. Pyzer-Knapp, *arXiv.org* **2018,** 1807.01279.

[2] Z. Sands, B. Mathieu, A. Valade, E. Jnoff, A. Ates, P. Burssens, D. Skolc, *WIPO* **2016,** WO/2016/055479.

[3] Z. Hyder, J. Ruan, J. Xiao, *Chem. Eur. J.* **2008,** *14*, 5555-5566.
